# Complete Genome Sequence of the Highly Pathogenic Strain A/Domestic Goose/Pavlodar/1/05 (H5N1) of the Avian Influenza Virus, Isolated in Kazakhstan in 2005

**DOI:** 10.1128/MRA.00109-20

**Published:** 2020-03-05

**Authors:** Aisha Issabek, Yerbol Burashev, Olga Chervyakova, Mukhit Orynbayev, Zhailaubay Kydyrbayev, Markhabat Kassenov, Kunsulu Zakarya, Kulyaisan Sultankulova

**Affiliations:** a Research Institute for Biological Safety Problems (RIBSP), Gvardeyskiy, Kazakhstan; Queens College

## Abstract

Here, we present the complete genome sequence of a highly pathogenic strain of avian influenza A virus/domestic goose/Pavlodar/1/05 (H5N1) (GS/1/05), which belongs to clade 2.2. This strain of the influenza virus was isolated in northern Kazakhstan in 2005.

## ANNOUNCEMENT

Influenza A virus belongs to the family *Orthomyxoviridae*. Influenza A virus exists in many different environments and infects different types of hosts (1). The ecology of these viruses varies greatly depending on their interaction with each other (reassortment, competition), with their hosts (immunity, receptor availability, host temperature), and with the environment (ambient temperature, humidity, sediment composition, water salinity, pH) (2). The segmented genome of influenza A virus provides evolutionary benefits. Influenza A virus has a great ability to evolve using two different mechanisms, antigenic drift and shift. All pandemics in the 20th century were of avian origin (3, 4).

Field expeditions were organized to the Kazakhstan regions with active migration of wild birds and large populations of domestic birds. The domestic birds were caught manually. A cloacal swab sample was taken from the domestic birds at the time of capture, and the birds were immediately returned to the flock. A smear from the cloaca was taken with a swab, after which the swab was put into a cryovial with a transport medium (bovine infusion broth, bovine albumin fraction V, gentamicin sulfate, and amphotericin B [Fungizone]), and the cryovial was immediately placed in liquid nitrogen. Cryovials with a transport medium were kept in liquid nitrogen or a refrigerator at 40°C until work began. Field materials were transported in Dewar’s vessels with liquid nitrogen. In the laboratory, samples were stored at –70°C before the study.

Viral RNA was extracted using the QIAamp viral RNA extraction kit (Qiagen) according to the manufacturer’s instructions. All eight segments were amplified using SuperScript one-step reverse transcriptase PCR (RT-PCR) kits with Platinum *Taq* (Invitrogen SRL). Sequencing and amplification of eight segments of the virus genome were done with the Uni-12 (3′-UCG YUU UCG UCC) and Uni-13 (GG AAC AAA GAU GA-5′) universal primers (5). Sequencing was performed with a 16-capillary genetic analyzer AB3130xl automatic sequencer (Hitachi Applied Biosystems) using the BigDye Terminator version 3.1 cycle sequencing kit (ABI, Foster City, CA, USA). Chromatograms were edited using Sequencer version 5 (Gene Codes Corp.) and BioEdit version 7.2.5 (http://www.mybiosoftware.com/alignment/1013) for sequence assembly and alignment. Genome sequencing of avian influenza A virus/domestic goose/Pavlodar/1/05 (GS/1/05) yielded sequences of all eight genomic segments, including polymerase basic 2 (PB2), polymerase basic (PB1), polymerase acidic (PA), hemagglutinin (HA), nucleoprotein (NP), neuraminidase (NA), matrix (M), and nonstructural (NS). As a result of the laboratory analysis, strain GS/1/05 was isolated. For virus isolation, the sample was passaged twice on 10-day-old chicken embryos obtained from local poultry farms that are safe from infectious diseases and do not have an immune background to type A influenza viruses. Based on genetic analysis of HA (site of proteolytic cleavage), NA (presence of a deletion of 20 amino acids), and NS1 (presence of a deletion of 5 amino acids) pathogenicity factors, GS/1/05 was assigned to a highly pathogenic virus strain, avian influenza (HPAI H5N1), having an increased tropism for mammalian cells and resistance to the antiviral effects of interferons and tumor necrosis factor. The size of each obtained viral segment is shown in Table 1. A BLAST search in the GenBank database showed that each of the eight isolated genes shared a high nucleotide identity with viruses isolated from birds in 2005 to 2006.

**TABLE 1:** Genome characteristics of strain A/domestic goose/Pavlodar/1/05

Gene/segment	Size (nucleotides)	GC content (%)^*a*^	Strain with closest relative	Identity at nucleotide level (%)	GenBank accession no.
PB2	2,326	44.3	A/chicken/Omsk/14/05(H5N1)	99.74	EF205204
PB1	2,298	42.6	A/Bar-headed Goose/Qinghai/62/05(H5N1)	99.83	DQ095740
PA	2,216	43.7	A/turkey/Suzdalka/12/05(H5N1)	99.55	EF205189
HA	1,735	41.0	A/cygnusolor/Croatia/1/2005(H5N1)	99.25	CY016819
NP	1,565	47.5	A/goose/Krasnoozerskoe/627/05(H5N1)	99.62	EF205178
NA	1,324	44.0	A/goose/Suzdalka/10/05(H5N1)	99.85	EF205170
M	1,006	47.6	A/Anas platyrhynchos/ Slovenia/359/06(H5N1)	99.50	AM911098
NS	885	43.3	A/goose/Krasnoozerskoe/627/05(H5N1)	99.66	EF205185

a Nucleotide sequence identities between the GS/1/05 strain and the closest homologs in the GenBank database.

**FIG 1:**
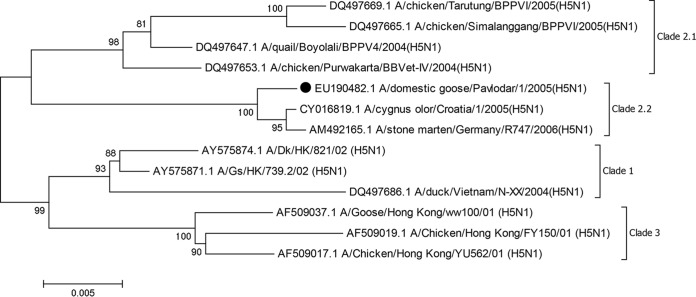
Phylogeny of the HA gene was inferred using the maximum likelihood method with 1,000 bootstrap replicates. At each branch, the number indicates a bootstrap value (>70%). The black circle indicates the A/domestic goose/Pavlodar/1/05 (H5N1) virus.

The phylogenetic tree at the nucleotide level for the hemagglutinin gene was constructed using the Tamura 3-parameter model (6) in MEGA version 7.0 (7). According to phylogenetic analysis, strain GS/1/05 is included in the Qinghai-Siberian group (clade 2, subclade 2) (Fig. 1).

### Data availability.

The complete genome sequence of strain A/domestic goose/Pavlodar/1/05 (H5N1) has been deposited in GenBank under the accession numbers EU213048 to EU213051, EU190482, and EU213068 to EU213070.
